# Global Transcriptomic Analyses Reveal Genes Involved in Conceptus Development During the Implantation Stages in Pigs

**DOI:** 10.3389/fgene.2021.584995

**Published:** 2021-02-24

**Authors:** Xupeng Zang, Ting Gu, Qun Hu, Zhiqian Xu, Yanshe Xie, Chen Zhou, Enqin Zheng, Sixiu Huang, Zheng Xu, Fanming Meng, Gengyuan Cai, Zhenfang Wu, Linjun Hong

**Affiliations:** ^1^National Engineering Research Center for Breeding Swine Industry, College of Animal Science, South China Agricultural University, Guangzhou, China; ^2^Guangdong Provincial Key Laboratory of Agro-Animal Genomics and Molecular Breeding, College of Animal Science, South China Agricultural University, Guangzhou, China; ^3^Lingnan Guangdong Laboratory of Modern Agriculture, Guangzhou, China; ^4^Institute of Animal Science, Guangdong Academy of Agricultural Sciences, Guangzhou, China

**Keywords:** pig, conceptus, implantation, development, RNA-Seq

## Abstract

Prenatal mortality remains a significant concern to the pig farming industry around the world. Spontaneous fetal loss ranging from 20 to 45% by term occur after fertilization, with most of the loss happening during the implantation period. Since the factors regulating the high mortality rates of early conceptus during implantation phases are poorly understood, we sought to analyze the overall gene expression changes during this period, and identify the molecular mechanisms involved in conceptus development. This work employed Illumina’s next-generation sequencing (RNA-Seq) and quantitative real-time PCR to analyze differentially expressed genes (DEGs). Soft clustering was subsequently used for the cluster analysis of gene expression. We identified 8236 DEGs in porcine conceptus at day 9, 12, and 15 of pregnancy. Annotation analysis of these genes revealed rRNA processing (GO:0006364), cell adhesion (GO:1904874), and heart development (GO:0007507), as the most significantly enriched biological processes at day 9, 12, and 15 of pregnancy, respectively. In addition, we found various genes, such as T-complex 1, RuvB-like AAA ATPase 2, connective tissue growth factor, integrins, interferon gamma, SLA-1, chemokine ligand 9, PAG-2, transforming growth factor beta receptor 1, and Annexin A2, that play essential roles in conceptus morphological development and implantation in pigs. Furthermore, we investigated the function of PAG-2 *in vitro* and found that PAG-2 can inhibit trophoblast cell proliferation and migration. Our analysis provides a valuable resource for understanding the mechanisms of conceptus development and implantation in pigs.

## Introduction

The implantation period is critical for the development of conceptuses during the early stages of pregnancy in swine. Previous studies have reported over 30% prenatal mortality rates of pig conceptus, after ovulation and fertilization, especially at gestation day 11 to 13 (Zavy and Geisert, 1994). Unlike other animals, porcine conceptuses undergo a unique rapid transformation in morphology, between days 10 and 12 of gestation, mainly due to extensive cellular migration and reorganization (Mattson et al., 1990). These transformations are mainly manifested as changes in conceptus size and shape, and range from spherical (3 to 10 mm diameter) to tubular (10 to 50 mm long) to filamentous (>100 mm long; Perry and Rowlands, 1962; Anderson, 1978). At gestation day 15, filamentous conceptuses extend from 800 to 1000 mm in length, and start to attach to the uterine luminal epithelium (Bazer and Johnson, 2014). Elongation in the uterine horns, before conceptus implantation, simultaneously create a larger contact between the conceptus and maternal tissues, which is crucial for subsequent conceptus attachment (Keys et al., 1986; Bazer and Johnson, 2014).

Studies have implicated rapid conceptus elongation in the increase in conceptus mortality, and this follows a specific pattern of gene expression (Niemann and Wrenzycki, 2000). During this period, estrogens synthesized, and released by the conceptus appear and are considered signals for maternal recognition of pregnancy (Flint et al., 1979). Furthermore, many endometrial genes such as progesterone, glucocorticoids, prostaglandins, and interferons, that could affect uterine receptivity to implantation in pigs are stimulated by estrogens (Bazer et al., 2009). Besides, several cytokines, released by the porcine conceptus, have been implicated in the induction of physiological changes to their corresponding receptors thereby playing an important role in regulating cell proliferation, movement, adhesion, as well as establishment of the microenvironment of the uterine cavity immune response and attachment of trophectoderm to the uterine luminal epithelium (Bazer et al., 2009; Geisert et al., 2017). Although some work has been done on this front, dynamic changes during the implantation process are not fully understood necessitating a comprehensive transcriptome analysis.

In this study, we sought to understand the molecular mechanisms of conceptus development and attachment, by analyzing and comparing expression profiles of mRNAs in the porcine conceptuses across different phases of implantation (days 9, 12, and 15) using RNA-Seq technology. We identified differentially expressed genes (DEGs) at the aforementioned time points and performed a functional analysis of these genes using bioinformatic tools. In addition, we substantiated previous findings of the potential importance of PAG-2 for trophoblast cell proliferation and migration. Collectively, these results generate a better understanding of the genetic factors regulating conceptus development during the implantation stages.

## Materials and Methods

### Animals and Conceptus Collection

This study was approved by the Ethics Committee of the Laboratory Animal Center of South China Agricultural University. Yorkshire sows (parity 2) were obtained from the Wen’s Foodstuffs Group Co., Ltd. (Yunfu, China). The sows were checked for estrus, twice a day, then artificially inseminated with a standard dose of single Yorkshire semen following estrus. The sows were sacrificed on days 9, 12, and 15 of pregnancy (*n* = 3 sows/day of pregnancy), their uteri immediately removed, and each uterine horn flushed with bacteria-free PBS. The conceptuses were then collected, snap frozen, and stored at −80°C for RNA extraction.

### Construction of mRNA Libraries and Sequencing

Total RNA was extracted from each sample using TRIzol Reagent (Invitrogen, Carlsbad, CA, United States) according to the manufacturer’s protocol. The RNA was quantified and qualified using an Agilent 2100 Bioanalyzer (Agilent Technologies, Palo Alto, CA, United States) and NanoDrop (Thermo Fisher Scientific, Wilmington, MA, United States). Then, 1 μg of the total RNA, with a RIN value above 7, was used for the construction of libraries according to the manufacturer’s instructions using the NEBNext^®^ Ultra^TM^ Directional RNA Library Prep kit (Illumina^®^). The Ribo-Zero^TM^ rRNA Removal Kit (Illumina, San Diego, CA, United States) was used to deplete rRNA from total RNA. The pure RNA was then fragmented and reverse-transcribed, then libraries with different indices were multiplexed and loaded on an Illumina HiSeq 150PE (Anders et al., 2015) instrument according to the manufacturer’s instructions (Illumina, San Diego, CA, United States). Sequencing was performed using the paired-end (PE) configuration on the HiSeq instrument at the GENEWIZ (Suzhou, China) to generate 2 × 150 bp transcripts.

### Analysis of RNA-Seq Data

Raw reads were first processed using Cutadapt (v1.3; Martin, 2011) before mapping and assembly. In this step, clean reads were acquired by removing adapter sequences, as well as nucleotides with *q*-quality scores lower than 20 or bases with N, and reads less than 75 bp after trimming. All subsequent downstream processes were performed using high-quality clean data.

### Estimation of Transcript Abundance and Identification of DEGs

Aligned read files were processed by Hisat2 (v2.0.1; Daehwan et al., 2015), which uses fragment per kilobase of exon per million fragments mapped (FPKM) to measure relative abundances of the transcripts. The reference GFF annotation file (Sscrofa10.2, http://may2017.archive.ensembl.org/Sus_scrofa/Info/Index), used in Hisat2, was downloaded from the ENSEMBL database, then StringTie (v1.0.4; Mihaela et al., 2015) was used to first assemble the transcriptome. Cuffmerge (Trapnell et al., 2012) was then adopted to merge all transcripts from different replicas in a group and generate unique transcripts for further downstream differential expression analysis. Sequence information corresponding to these transcripts was obtained using Gffread (Trapnell et al., 2010). Differential gene expression analyses among samples were conducted using the DESeq tool (v1.18.0; Love et al., 2014), which is a model based on the negative binomial distribution. We performed adjustment using the Benjamini and Hochberg (1995)’s approach to control for false discovery rate, with a *P* < 0.01 and | log2 (fold change) | >1, for the detection of DEGs.

### Quantitative Real-Time PCR

We validated the gene expression profiles across the study groups using quantitative real-time PCR (qRT-PCR). Total RNA was first extracted using the RNeasy Plus Micro Kit (Qiagen, Hilden, Germany) according to the manufacturer’s instructions, then one microgram of the RNA was reverse transcribed into cDNA using the PrimeScript^TM^ RT Master Mix kit (TakaRa, Dalian, China) to generate a template for PCR. qPCR was carried out on the QuantStudio 7 Flex Real-Time PCR System (Applied Biosystems, Foster city, CA, United States) using a PowerUpTM SYBRTM Green Master Mix (Thermo Fisher, United States). The reaction (10 μL) comprised 5 μL of PowerUpTM SYBRTM Green Master Mix, 2 ng/μL of cDNA, and 0.2 μM of each primer and nuclease-free water. Amplification conditions included an initial step at 94°C for 5 min, followed by 40 cycles of 94°C for 30 s, an annealing step of 60°C for 20 s, extension at 72°C for 30 s, and a final extension of 4 min at 72°C. Primers were designed by Oligo 7 (Molecular Biology Insights Inc., Colorado Springs, United States), and their sequence specificities were checked using BLAST (NCBI, Bethesda, United States). A summary of primers and their corresponding annealing temperatures is outlined in Supplementary Table 3. Analysis was performed on three biological replicates for each time-point, with three technical replicates. In addition, GAPDH was used as the internal amplification control.

### Analysis of DEGs and Differentially Co-Expressed Genes

Hierarchical cluster analysis was used to assess differential expression among genes across the three stages of pregnancy. Differentially co-expressed genes were obtained from the overlap of DEGs in three stages. To expose the functions of these differentially co-expressed genes, gene ontology (GO) enrichment analysis was performed by the software DAVID (v6.8)^1^ andKyoto Encyclopedia of Genes and Genomes (KEGG) pathway analysis was executed by the software KOBAS^2^. Soft clustering, using the Mfuzz package implemented in R (Kumar and Futschik, 2007), was then performed to obtain the DEGs and reveal their expression profiles based on similar expression patterns. Transcript IDs of the DEGs from individual clusters were uploaded onto DAVID (v6.8; see text footnote 1) and used for GO analyses (Dennis et al., 2003). DEGs from various clusters were also screened using the online tool Toppcluster^3^ for KEGG pathway analysis, with a *p*-value cutoff of 0.05. Then Cytoscape (v3.7.2)^4^ was used to visualize the final results (Shannon et al., 2003; Kaimal et al., 2010).

### Cell Culture and Transfection

The cell culture was performed as described in our previous studies (Hong et al., 2020). The small interference RNAs (siRNAs) for PAG-2 were from GenePharma (Shanghai, China). Full-length PAG-2 was amplified and inserted into the pcDNA3.1 vector (Invitrogen, Carlsbad, CA, United States) to construct a plasmid and transiently transfected with Lipofectamine 2000 (Invitrogen, Carlsbad, CA, United States) according to the manufacturer’s instructions.

### Cell Counting Kit-8 Assay

PTr2 cells were seeded in 96-well plates (10,000 cells/well). After the cells were transfected for 48 h and 72 h, 10 μl of Cell Counting kit-8 (CCK-8) solution was added to the wells. The plates were incubated for 1 h, and the absorbance of each well was collected by a microplate reader (Tecan, Switzerland) at 450 nm.

### 5-Ethynyl-2’-Deoxyuridine Assay

PTr2 cells were seeded in 24-well plates (50,000 cells/well), and after being cultured overnight, they were transfected with pcDNA3.1(+), pcDNA3.1(+)-PAG-2, siRNA-NC, or siRNA-PAG-2. After transfection for 48 h and 72 h, PTr2 cells were exposed to EdU (BeyoClick, China) for 3 h at 37°C. Subsequently, the cells were fixed in 4% paraformaldehyde for 15 min, were wiped with washing solution, and then permeabilized by adding 0.3% Triton X-100. Next, plates were washed with PBS, 0.3 ml of Click was added and the cells were incubated at room temperature in the dark for 30 min. The nuclear stain DAPI was then added, and a confocal laser scanning microscope (Leica, Germany) was used to photograph and visualize the number of EdU-stained cells.

### Wound Healing Assay

PTr2 cells were seeded in 6-well plates and cultivated until they reached confluence. Wounds were generated in the cell monolayer by making a scratch with a sterile pipette tip. Plates were washed three times with PBS, and then added to serum-free medium and cultured in a 37°C 5% CO2 cell incubator. The plates were photographed after 0 and 24 h of cultivation.

### Transwell Migration and Invasion Assay

Approximately 60,000 PTr2 cells were suspended in serum-free medium and seeded in the upper chambers with 8 μm (Corning, New York, NY, United States). For this assay, medium containing 10% FBS was added to the lower chambers and incubated for a further 24 h. The non-migrated or non-invaded cells were then wiped with PBS. The cells that had invaded through the membrane to the lower surface were fixed with 4% paraformaldehyde for 10 min and followed by crystal violet staining for 10 min. Then, random fields were photographed and counted by using a light microscope.

### Statistical Analysis

GraphPad Prism 8.0 (GraphPad Software, San Diego, CA, United States) was used to statistically analyze the transfection efficiency of PAG-2 overexpression or inhibition in PTr2 cells, the cell viability through CCK-8 assays, the cell proliferation rate of the 5-Ethynyl-2’-Deoxyuridine (EDU) staining assays, and the cell migration rate of the wound healing and Transwell assays. The values are presented as the mean ± standard error of mean (SEM). Where applicable, student’s *t*-tests were performed to test the statistical significance of the data. *P* < 0.05 was considered to be statistically significant, and *P* < 0.01 was considered to be extremely significant.

## Results

### RNA-Seq Data for the Porcine Conceptus During Days 9, 12, and 15 of Pregnancy

We generated RNA sequence data at 9, 12, and 15 of pregnancy and performed a transcriptomics analysis to comprehensively understand changes in expression levels across these time points. Summarily, we obtained 79,500,512 to 112,359,702 quality-filtered reads from 79,756,340 and 112,640,612 raw reads per sample, respectively (Table 1). We used Hisat2 (v2.0.1) to index reference genome sequences, and aligned 85.3076–86.2598% of reads to genome sequences at ENSEMBL, whereof 74.602–76.4987% of reads had a unique genomic site. Thereafter, a total of 547,885 transcripts were assembled using Cuffcompare.

**TABLE 1:** Summary of RNA-Seq alignment.

Sample	Raw reads	Clean reads	Clean data	Mapped reads	Unique mapped reads	Multi mapped reads	Pair-end mapped reads
D9-1	80264870	79920206	79920206	68178049	59622092	8555957	55462262
D9-2	99983254	99744758	99744758	85959081	75870870	10088211	71456836
D9-3	112640612	112359702	112359702	96921221	85014782	11906439	79691222
D12-1	79756340	79500512	79500512	68207012	60816876	7390136	56550554
D12-2	90024798	89795156	89795156	76819268	68087133	8732135	64065452
D12-3	82742908	82518014	82518014	70522524	62567425	7955099	58785092
D15-1	101114958	100851876	100851876	86841361	76739965	10101396	72360248
D15-2	99697522	99456902	99456902	85586127	75673496	9912631	71276362
D15-3	101700048	101442704	101442704	87219987	77018064	10201923	72659846

**FIGURE 1:**
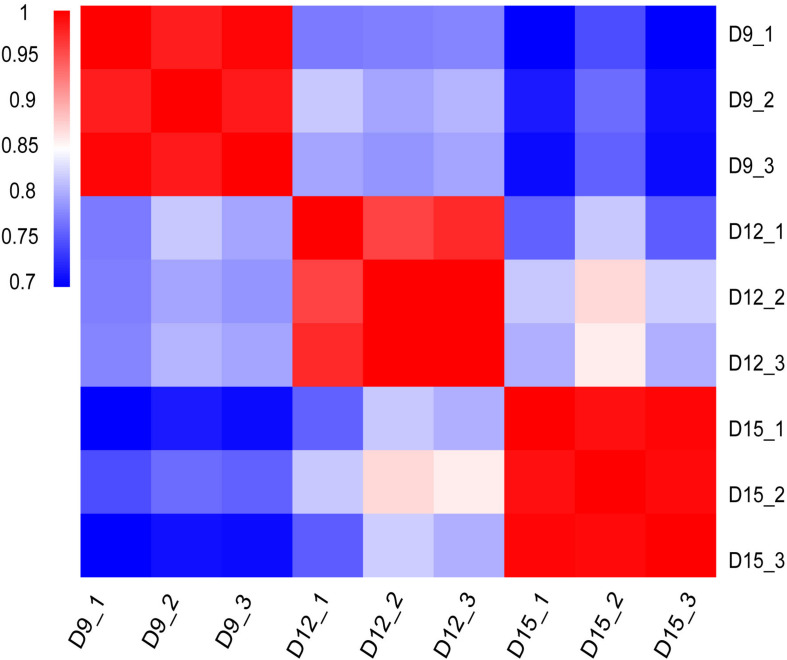
Matrix of Pearson’s correlation coefficient among transcriptomes across three different porcine conceptus phases on days 9, 12, and 15 of pregnancy. The color scale is from 0.7 (blue) to 1.0 (red). Red represents high correlation between biological repeats.

Distribution and identity of the three triplicate samples were analyzed by correlation, with Pearson’s correlation coefficient among transcriptomes across porcine conceptus phases on days 9, 12, and 15 revealing a good correlation of biological replicates (Figure 1). A summary of Pearson’s correlation coefficients is shown in Supplementary Table 1.

### DEGs Across the Phases During the Implantation Periods

**FIGURE 2:**
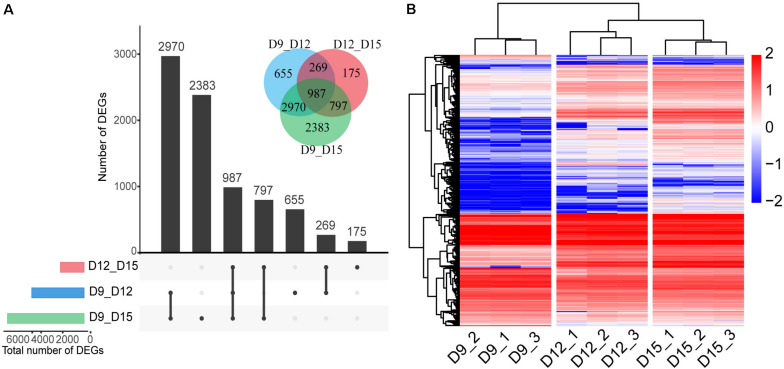
Differentially expressed genes (DEGs) of the porcine conceptus across three periods of pregnancy. Upset plot and Venn diagram illustrating the number of DEGs across the three developmental phases **(A)**. Red, day 12 vs. day 15 of conceptus; blue, day 9 vs. day 12 of conceptus; and green, day 9 vs. day 15 of conceptus. Hierarchical cluster analysis of DEGs identified for three phases **(B)**. The color scale is from -2.0 (blue, lower gene expression) to 2.0 (red, higher gene expression). Each row represents one gene, each column represents one sample.

We generated 848 million PE reads of 150 bp in length. Thus, the depth of our sequencing was adequate for detecting transcripts expressed at low levels. To identify the DEGs across different implantation phases, we performed comparisons between transcripts at days 9 and 12, 9 and 15, and 12 and 15. The analysis revealed a total number of 4881 DEGs between days 9 and 12, and 2228 between days 12 and 15, and 7137 between days 9 and 15 (FDR < 0.01, log2 fold change > 1; Figure 2A). The total number of DEGs across all comparisons (8236) was used for analysis of normalized expression profiles across the three developmental phases. Hierarchical cluster analysis was used to present an overview of the expression profiles, with all DEGs showing lower or higher expression during the three phases of pregnancy (Figure 2B and Supplementary Table 2). Results of the 9-sample dataset indicated that the gene expression pattern was more similar from days 12 to 15 than days 9 to 12, although the biggest difference in gene expression was observed between days 9 and 15 of pregnancy.

### Validation of RNA Sequence Data

**FIGURE 3:**
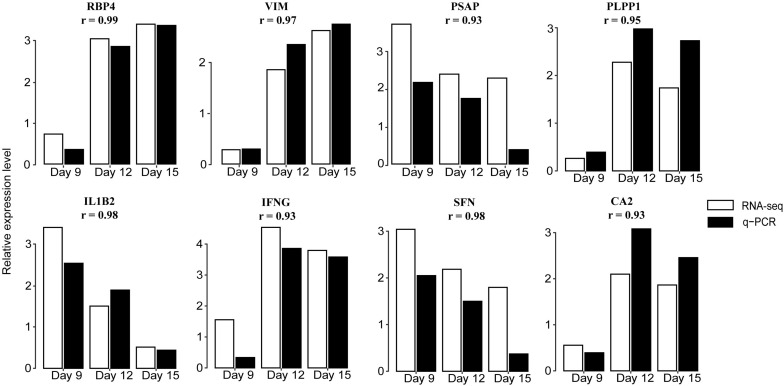
Validation of DEGs by qRT-PCR. Expression patterns of eight DEGs on days 9, 12, and 15 of pregnancy were determined by qRT-PCR and compared with profiles obtained by RNA-seq. White bars indicate RNA-seq expression level; black indicate q-PCR expression level. The correlation coefficient r represents a correlation between q-PCR and RNA-seq-based gene expression. Detailed results are shown in Supplementary Table 4. RBP4: retinol binding protein 4; VIM: vimentin; PSAP: prosaposin; PLPP1: phospholipid phosphatase 1; IL1B2: interleukin 1, beta 2; IFNG: interferon gamma; SFN: stratifin; and CA2: carbonic anhydrase 2.

To validate the RNA-seq data we performed a qRT-PCR analysis targeting eight genes, RBP4, VIM, PSAP, IL1B2, interferon gamma (IFNG), SFN, PLPP1, and CA2. These were randomly selected because they were significantly expressed in at least one contrast. Results showed good concordance in expression patterns determined by qRT-PCR and RNA-seq (Figure 3 and Supplementary Table 4).

### GO and KEGG Enrichment Analysis of Differentially Co-Expressed Genes

**FIGURE 4:**
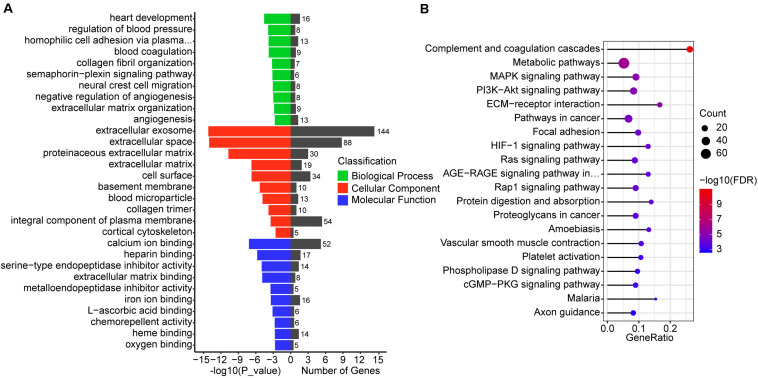
GO and KEGG enrichment analysis of differentially co-expressed genes. **(A)** GO enrichment analysis of differentially co-expressed genes, the terms include biological process, cellular component, and molecular function. **(B)** Top 20 KEGG pathways of differentially co-expressed genes. The size of the circle represents the number of genes enriched in the pathway and red represents more significant enrichment.

To understand the function of 987 differentially co-expressed genes in the three stages, we performed GO enrichment and KEGG pathways analyses. The top 10 enriched GO terms in biological process, cellular component, and molecular function are listed in Figure 4A, while all the GO enrichment terms are shown in Supplementary Table 5. Notably, these biological processes were mostly associated with angiogenesis, such as regulation of blood pressure (GO:0008217), blood coagulation (GO:0007596), negative regulation of angiogenesis (GO:0016525), and angiogenesis (GO:0001525), which indicates the violent development of the vascular system during conceptus development at these stages. KEGG pathway analysis showed that several pathways were related to signal transduction, such as the MAPK signaling pathway, PI3K-Akt signaling pathway, HIF-1 signaling pathway, and Ras signaling pathway (Figure 4B and Supplementary Table 5).

### Relationship Between Gene Clusters and Biological Processes and Pathways

**FIGURE 5:**
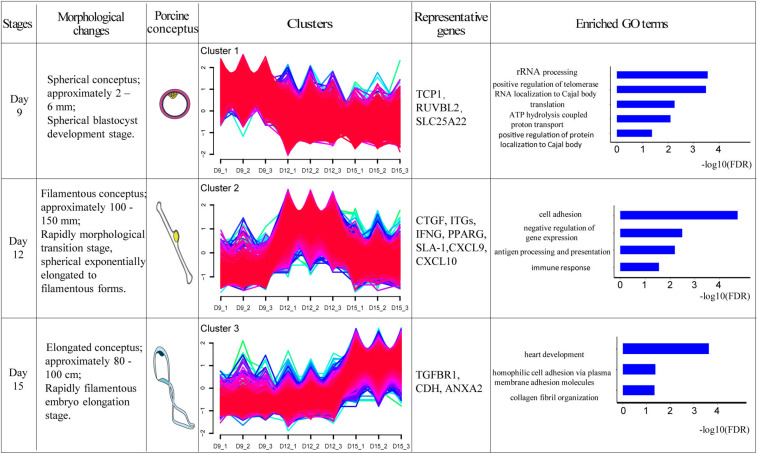
Transcriptome dynamics during porcine conceptus implantation development. The DEGs were clustered into three soft partitioning clusters, with distinct clusters falling into different morphological stages based on expression patterns. GO enrichment was then performed on each cluster of genes and the GO term represented was displayed (FDR < 0.05). Morphological changes briefly described the development of conceptus at this stage. Porcine conceptus represented the pattern diagrams of conceptus. TCP1: T-complex 1; RUVBL2: RuvB-like AAA ATPase 2; SLC25A22: solute carrier family 25 member 22; CTGF: connective tissue growth factor; ITGs: integrins, IFNG: interferon gamma; PPARG: peroxisome proliferator activated receptor gamma; SLA-1: MHC class I antigen 1; CXCL9: chemokine ligand 9; CXCL10: chemokine ligand 10; TGFBR1: transforming growth factor beta receptor 1; CDH: cadherin 11; and ANXA2: Annexin A2.

**FIGURE 6:**
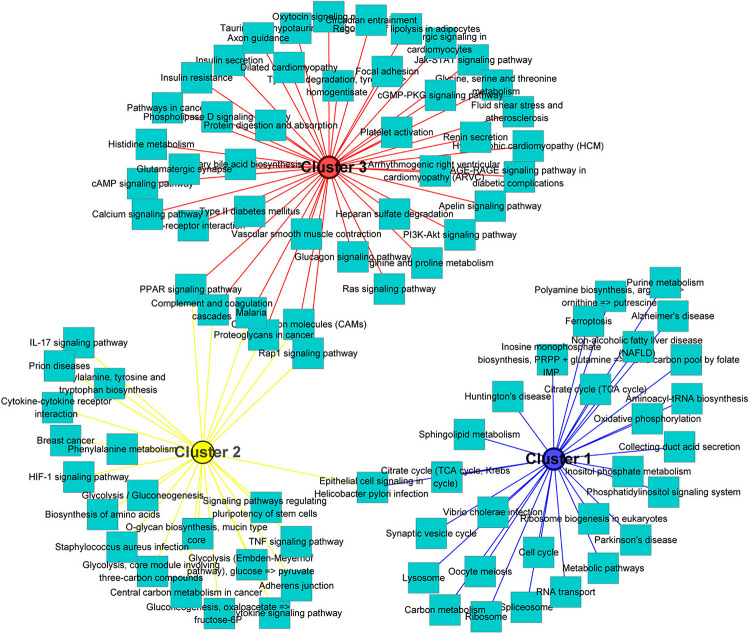
KEGG pathways analysis of gene clusters. All significant differentially expressed genes across the three stages were used as input for the ToppCluster. The database “KEGG pathway” was used for analysis. Then, the data were uploaded in Cytoscape (v3.7.2) to generate the network. Each square node represents a pathway. Each cluster is represented by a different color.

One of the aims of this study was to analyze the relationship between morphological and potential molecular changes during the implantation development of pig conceptus. Consequently, we used the soft partitioning clustering method of the Mfuzz package to generate three visible clusters of genes that were distributed across the three implantation stages according to their analogical expressions pattern. The first cluster showed the highest expression levels on day 9, although this was rapidly down-regulated. The second cluster exhibited the highest expression levels on day 12, whereas the last cluster showed specific highest expression levels on day 15 (Figure 5). Each gene cluster was then subjected to GO enrichment analysis (only GO terms related to biological processes were considered) to determine whether identifiable biological changes were significantly related to expression patterns. A detailed outline of the results is presented in Supplementary Table 6. Summarily, genes from the first cluster were strongly associated with rRNA processing (GO:0006364), positive regulation of telomerase RNA localization to Cajal body (GO:1904874), translation (GO:0006412), ATP hydrolysis-coupled proton transport (GO:0015991), and positive regulation of protein localization to Cajal body (GO:1904871). Representative genes in these biological processes comprised T-complex 1 (TCP1), RuvB-like AAA ATPase 2 (RUVBL2), and solute carrier family 25 member 22 (SLC25A22). On the other hand, genes in the second cluster were strongly associated with cell adhesion (GO:0007155), negative regulation of gene expression (GO:0010629), antigen processing and presentation (GO:0019882) as well as immune response (GO:0006955). Lastly, genes in the third cluster were associated with heart development (GO:0007507), homophilic cell adhesion via plasma membrane adhesion molecules (GO:0007156), and collagen fibril organization (GO:0030199). Additionally, KEGG pathway analysis of the identified DEGs using ToppCluster revealed that the PPAR signaling pathway, complement and coagulation cascades, cell adhesion molecules, and the Rap1 signaling pathway among others were significant pathways across the time points (Figure 6 and Supplementary Table 7).

### PAG-2 Inhibits Trophoblast Cell Proliferation and Migration

**FIGURE 7:**
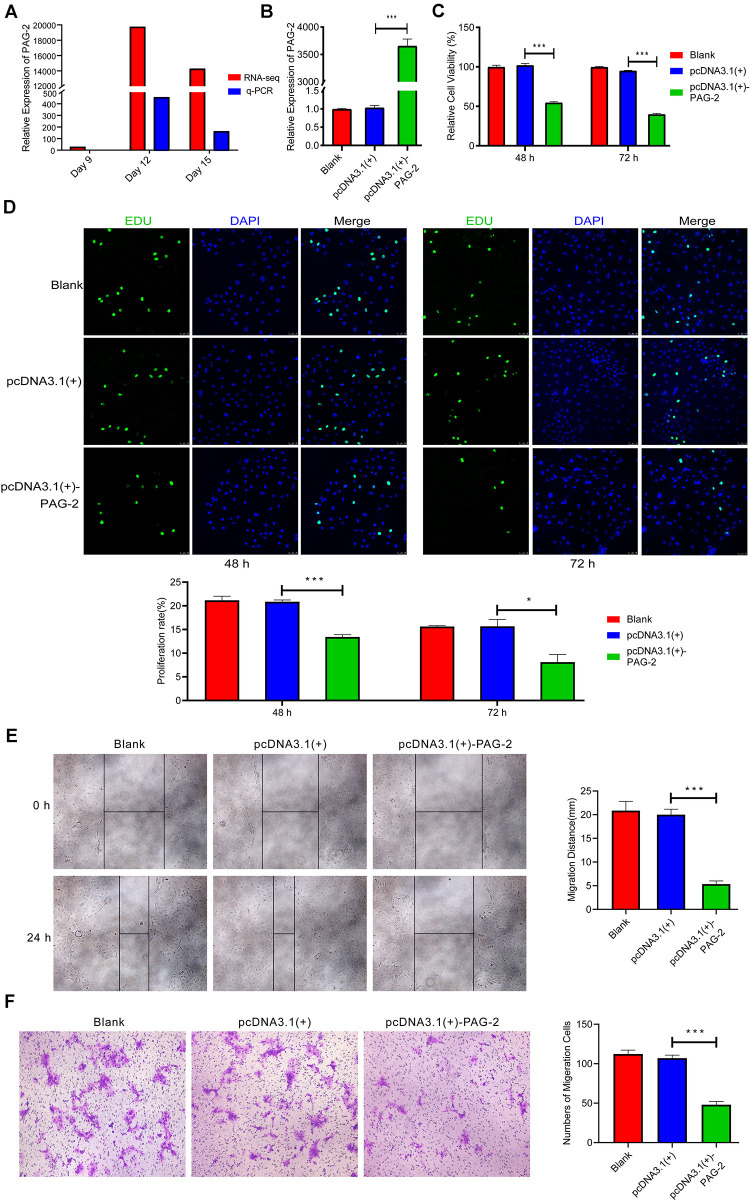
PAG-2 inhibits cell proliferation and migration *in vitro*. **(A)** The expression level of PAG-2 in porcine conceptus at days 9, 12, and 15 of pregnancy. **(B)** Transfection efficiency of PAG-2 overexpression was determined by PCR. **(C)** The cell viability of PTr2 cells was applied by CCK-8 assay. **(D)** EDU staining assay was performed to determine the cell proliferation changes after PAG-2 overexpression. **(E)** Wound healing assay for the evaluation of migration of PTr2 cells. **(F)** Transwell migration assay showed that PAG-2 overexpression reduced the cell numbers of migration. CCK8, Cell Counting Kit-8. Data are presented as mean ± SEM. *P < 0.05, ***P < 0.001, and Student’s *t*-test.

PAG-2 has been reported to have a role in the early pregnancy of water buffalo (Barbato et al., 2017), but its function during the implantation of pig conceptus has not been studied. In this study, PAG-2 was very highly up-regulated on day 12 of pregnancy, so it may play an important role during implantation (Figure 7A and Supplementary Table 2). Therefore, PAG-2 was selected as a candidate gene for regulating pig conceptus implantation.

**FIGURE 8:**
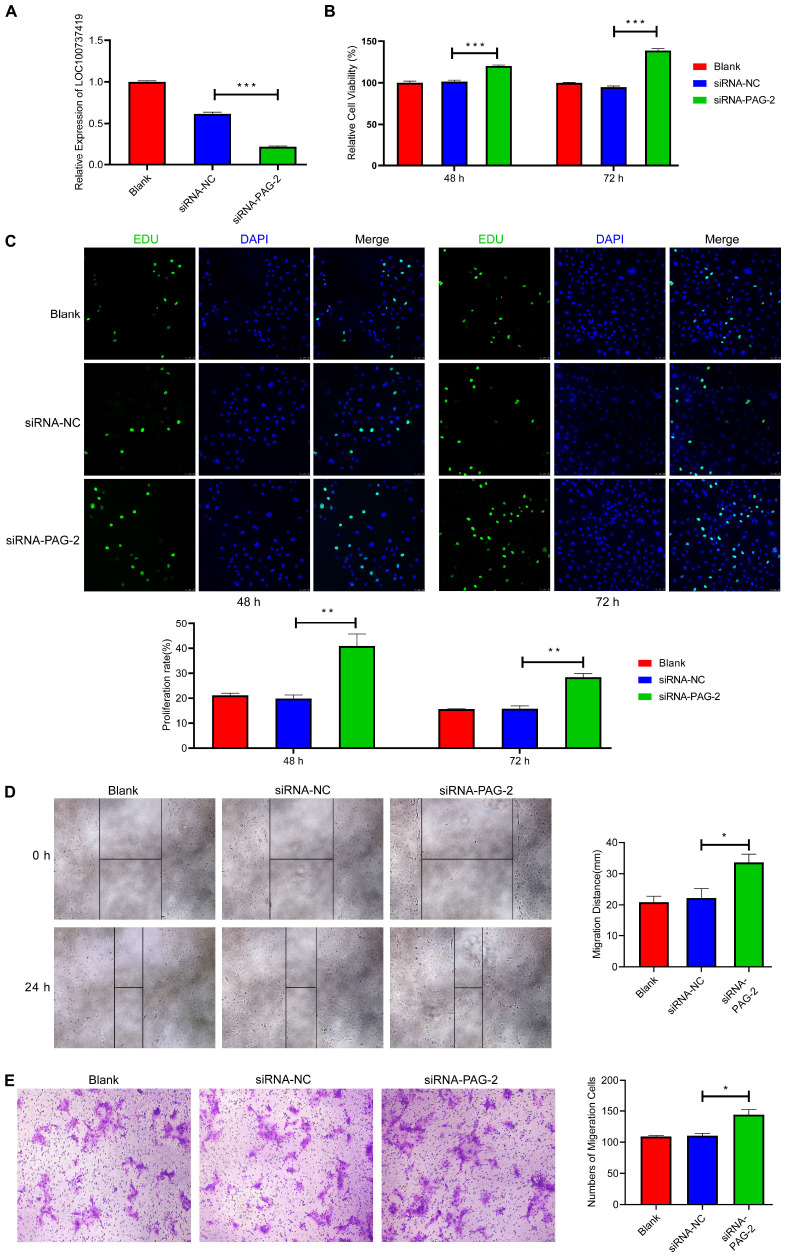
Knockdown of PAG-2 promotes cell proliferation and migration *in vitro*. **(A)** Transfection efficiency of PAG-2 siRNA was determined by PCR. **(B)** The cell viability of PTr2 cells was applied by CCK-8 assay. **(C)** EDU staining assay was performed to determine the cell proliferation changes after PAG-2 knockdown. **(D)** Wound healing assay for the evaluation of migration of PTr2 cells. **(E)** Transwell migration assay showed that knockdown PAG-2 increased the cell numbers of migration. CCK8, Cell Counting Kit-8. Data are presented as mean ± SEM. *P < 0.05, **P < 0.01, ***P < 0.001, and Student’s *t*-test.

To study the roles of PAG-2 on pig trophoblast cells, we conducted a series of *in vitro* experiments. Using the plasmid vector, we succeeded in overexpressing its level in PTr2 cells (Figure 7B). The CCK-8 assay showed that overexpression of PAG-2 significantly reduced trophoblast proliferation (Figure 7C). EDU staining also demonstrated that the proliferation rate of PAG-2 overexpression cells was significantly decreased compared with that of the control cells (Figure 7D). Wound healing migration and Transwell assays revealed that PAG-2 overexpression decreased PTr2 cells migration (Figures 7E,F). Conversely, the proliferation and migration of PTr2 cells were significantly promoted after PAG-2 knockdown (Figure 8). Together, the data suggest that PAG-2 can inhibit trophoblast cell proliferation and migration *in vitro*.

## Discussion

Recent studies have revealed that numerous genes play an important role in the development and loss of porcine conceptus, during early pregnancy stages (Franczak et al., 2018; Zeng et al., 2019). In the current study, we investigated gene expression patterns during early pregnancy stages after implantation and identified key genes that play important roles in successful implantation. A pig conceptus typically undergoes differentiation and expansion before attaching onto the surface of the maternal uterine epithelium. These rapid morphological changes can be attributed to changes in highly sophisticated and multifarious hormones and gene expression between days 9 and 12 of gestation.

On day 9 of pregnancy, porcine conceptus grows to spherical blastocysts, subsequently proliferates and differentiates through mitosis and slowly develops (Anderson, 1978). Our first gene cluster showed that the highest expression levels occurred on day 9, but this was rapidly down-regulated, indicating that genes in this cluster work mainly on day 9. GO enrichment analysis suggested that a majority of the highly expressed genes on day 9 were mainly related to rRNA processing (GO:0006364) and the positive regulation of telomerase RNA localization to Cajal body (GO:1904874). rRNA processing including cleavage, splicing, and other activities, has been reported to influence cell fate during early mammalian development (Corsini et al., 2018). On the other hand, positive regulation of telomerase RNA localization to Cajal body is known to exist in the cancer cell cycle, acting to promote the continuous proliferation of cells (Lingner et al., 1997; Zhu et al., 2004; Tomlinson et al., 2008; Musgrove et al., 2018). In addition, our GO results revealed enrichment of T-complex 1 (TCP1) and RuvB like AAA ATPase 2 (RUVBL2) for the positive regulation of telomerase RNA localization to Cajal body. Previous studies have shown that TCP1 encodes components of a multi-protein chaperone complex in the cells and promotes cell proliferation (Huang et al., 2014; Guest et al., 2015), whereas RUVBL2 has been found to function in cell cycle regulation (Venteicher et al., 2008; Jha and Dutta, 2009), and cell multiplication (Silva et al., 2018). Furthermore, RUVBL2 knockdown resulted in death of the conceptus (Arnold et al., 2012), indicating its important function for conceptus development. In our study, all these genes were highly expressed on day 9.

Day 12 of pregnancy represents a stage of exponential elongation for the porcine conceptus, from a spherical to filamentous state (Mattson et al., 1990). It has been hypothesized that genes up-regulated on day 12 (herein termed the second cluster) play an important role in the elongation stage. In the current study, we found significant enrichments of GO terms, mainly those related to cell adhesion (GO:1904874) and antigen processing as well as presentation (GO:0019882). Some genes known to be related to cell adhesion, including connective tissue growth factor (CTGF) and integrins (ITGs) were also observed. Previous studies have shown that CTGF regulates a wide range of biological activities. For instance, it has been found to play various roles in the uterus, including cell proliferation, differentiation, adhesion, chemotaxis, apoptosis, and angiogenesis (Lau and Lam, 1999; Perbal, 2001). This factor has also been implicated in endometrial extracellular matrix remodeling and angiogenesis during the critical period of conceptus attachment (Moussad et al., 2002). The extracellular matrix is composed of a complex mixture of structural and functional super molecules, such as collagens, laminins, and integrins (Bosman and Stamenkovic, 2003). Particularly, integrins, are repeatedly expressed and play a considerable role in the crosstalk between cells and extracellular matrix. On the other hand, the cell-matrix adhesion has been shown to cause cytoskeletal reorganization to stabilize adhesion, thereby regulating the attachment of trophectoderm to the uterine luminal epithelium (Albelda and Buck, 1990; Geisert et al., 2015). Moreover, this molecule has been predicted to be an important pathway for successful implantation (Wang et al., 2019).

Studies have shown that numerous immune reactions synchronously occur in the uterus during early pregnancy, to prevent immune rejection caused by the semi-allogeneic fetus and allow successful maternal recognition (Tayade et al., 2005; Trowsdale and Betz, 2006). Our results showed that genes that were highly expressed on day 12 were involved in conceptus development and maternal-uterine recognition, which is consistent with previous studies. For instance, we identified the antigen processing and presentation (GO:0019882) pathway, which has been shown to play a role in immunomodulation through a number of small proteins (Alessio et al., 2017), as well as IFNG and MHC class I antigen 1 (SLA-1). IFNG is a pro-inflammatory cytokine with extended roles in the activation of innate and adaptive immune responses, partly by up-regulating the transcription of genes involved in cell cycle regulation, apoptosis, and antigen processing/presentation (Murphy et al., 2009). It has been reported to positively influence the expression of some chemokines in endometrial cells, including chemokine ligand 9 (CXCL9) and chemokine ligand 10 (CXCL10), which are related to the recruitment of immune cells and the establishment of an immunotolerant environment (Złotkowska and Andronowska, 2019). Besides, expression of swine leukocyte antigen-DQ is induced by IFNG, and potentially regulates the immune response at the maternal-fetal interface to support the maintenance of pregnancy (Kim et al., 2012). On the other hand, SLA-1 is a polymorphic cell surface glycoprotein that binds to inhibitory and activating receptors on natural killer (NK) cells as well as other leukocytes (Davies et al., 2000). SLA-1 is specifically regulated by progesterone and IFNs, and provides an immunologically favorable environment for the survival of fetal-placental semi-allografts (Joyce et al., 2008).

In addition, PAG-2 is a member of the aspartic protease family and was originally found in the trophectoderm of fetal bovine (Xie et al., 1994), which was found to be associated with uterine immunosuppression during pregnancy (Serrano-Pérez et al., 2016). In this study, we found that PAG-2 was overexpressed on day 12 of pregnancy, and the experiments showed that it can inhibit the proliferation and migration of trophoblast cells. The elongation of the conceptus during the phase was mainly due to the remodeling of the trophoblast cells rather than proliferation, these results suggest that PAG-2 may play an important role in the process of implantation.

On day 15 of pregnancy, the filamentous conceptus further develops and begins to attach to the luminal uterine epithelium (LE; Bazer and Johnson, 2014). Our analysis revealed highly expressed genes on day 15, which play a crucial role in regulating conceptus development and attachment. For instance, we found enrichment of heart development (GO:0007507), homophilic cell adhesion via plasma membrane adhesion molecules (GO:0007156), and collagen fibril organization (GO:0030199) processes by genes specifically up-regulated on day 15 (the third cluster). Interestingly, no previous study has reported that a pig’s heart begins to develop on day 15 of pregnancy. However, our results support the hypothesis that conceptus heart may have started to develop on day 15 of pregnancy, which is meaningful for developmental biology research. Of the heart development genes identified herein, transforming growth factor beta receptor 1 (TGFBR1; GO:0007507), has been previously found to be expressed at the conceptus-maternal interface during early pregnancy stages in pigs (Jones et al., 2006). Functionally, this gene induces multiple cellular effects and has been shown to control proliferation, migration, and apoptosis, as well as promote conceptus development by increasing the proliferation of trophoblast cells (Roberts and Sporn, 1993; Blitek et al., 2013). In addition, we also identified Annexin A2, an essential gene for collagen fibril organization (GO:0030199). Previous studies have associated Annexin A2 with endometrial epithelial cell migration and trophoblast proliferation (Gerke and Moss, 1997), with its inhibition found to greatly reduce conceptus adhesion (Garrido-Gómez et al., 2012).

## Conclusion

In conclusion, our findings provide dynamic transcriptome changes during the implantation phases in pigs. Moreover, we revealed the function of PAG-2 on pig trophoblast cells *in vitro*. The genetic factors and pathways identified herein will be helpful to guide further research on developmental biology, human medicine, and increase livestock productivity.

## Data Availability Statement

The raw reads produced in this study were deposited in the NCBI Sequence Read Archive (SRA). The Accession number is PRJNA646603.

## Ethics Statement

The animal study was reviewed and approved by the Ethics Committee of the Laboratory Animal Center of South China Agricultural University.

## Author Contributions

XZ, TG, ZW, and LH developed and designed the research. QH, ZQX, YX, CZ, EZ, SH, ZX, and FM collected the samples and performed the experiments. XZ, TG, and GC performed the sequencing analysis and drafted the manuscript. All authors read and approved the final version of the manuscript.

## Conflict of Interest

The authors declare that the research was conducted in the absence of any commercial or financial relationships that could be construed as a potential conflict of interest.

## Footnotes

1
https://david.ncifcrf.gov

2
http://kobas.cbi.pku.edu.cn/kobas3

3
https://toppcluster.cchmc.org

4
http://www.cytoscape.org
