# Corrigendum: Global Transcriptomic Analyses Reveal Genes Involved in Conceptus Development During the Implantation Stages in Pigs

**DOI:** 10.3389/fgene.2021.721458

**Published:** 2021-08-05

**Authors:** Xupeng Zang, Ting Gu, Qun Hu, Zhiqian Xu, Yanshe Xie, Chen Zhou, Enqin Zheng, Sixiu Huang, Zheng Xu, Fanming Meng, Gengyuan Cai, Zhenfang Wu, Linjun Hong

**Affiliations:** ^1^National Engineering Research Center for Breeding Swine Industry, College of Animal Science, South China Agricultural University, Guangzhou, China; ^2^Guangdong Provincial Key Laboratory of Agro-Animal Genomics and Molecular Breeding, College of Animal Science, South China Agricultural University, Guangzhou, China; ^3^Lingnan Guangdong Laboratory of Modern Agriculture, Guangzhou, China; ^4^Institute of Animal Science, Guangdong Academy of Agricultural Sciences, Guangzhou, China

**Keywords:** pig, conceptus, implantation, development, RNA-Seq

In the original article, there was a mistake in Figure 8 as published. The images of siRNA-NC and siRNA-PAG-2 in Figure 8D were placed upside down. We found that the error was caused by our carelessness in typesetting. The corrected Figure 8 appears below.

**Figure 8:**
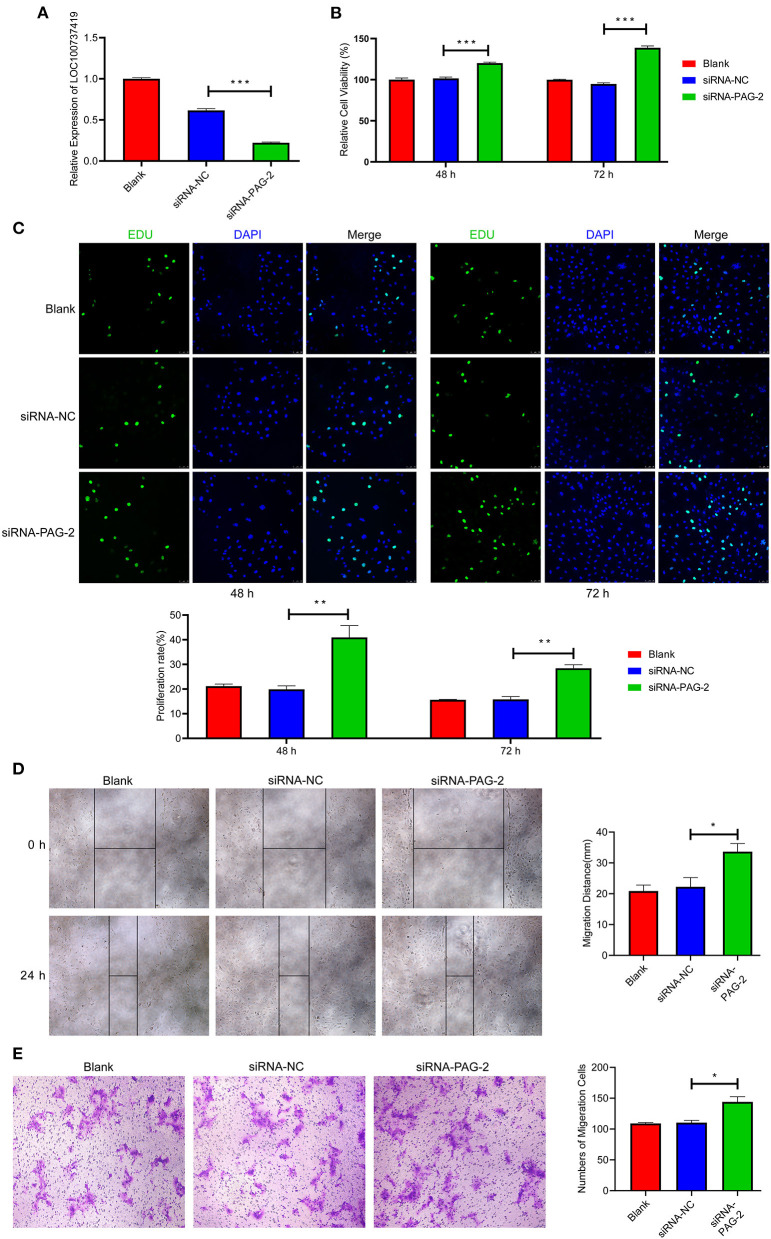
Knockdown of PAG-2 promotes cell proliferation and migration *in vitro*. **(A)** Transfection efficiency of PAG-2 siRNA was determined by PCR. **(B)** The cell viability of PTr2 cells was applied by CCK-8 assay. **(C)** EDU staining assay was performed to determine the cell proliferation changes after PAG-2 knockdown. **(D)** Wound healing assay for the evaluation of migration of PTr2 cells. **(E)** Transwell migration assay showed that knockdown PAG-2 increased the cell numbers of migration. CCK8, Cell Counting Kit-8. Data are presented as mean ± SEM. *P < 0.05, **P < 0.01, ***P < 0.001, Student's *t*-test.

The authors apologize for this error and state that this does not change the scientific conclusions of the article in any way. The original article has been updated.

## Publisher's Note

All claims expressed in this article are solely those of the authors and do not necessarily represent those of their affiliated organizations, or those of the publisher, the editors and the reviewers. Any product that may be evaluated in this article, or claim that may be made by its manufacturer, is not guaranteed or endorsed by the publisher.

